# Impact of functional inorganic nanotubes f-INTs-WS_2_ on hemolysis, platelet function and coagulation

**DOI:** 10.1186/s40580-018-0162-1

**Published:** 2018-10-30

**Authors:** Julie Laloy, Hélène Haguet, Lutfiye Alpan, Daniel Raichman, Jean-Michel Dogné, Jean-Paul Lellouche

**Affiliations:** 1 Namur Nanosafety Centre, University of Namur, Rue de Bruxelles 61, 5000 Namur, Belgium; 2 Department of Pharmacy, NARILIS, University of Namur, Namur, Belgium; 3 Department of Haematology Laboratory, Université catholique de Louvain, CHU UCL Namur, NARILIS, Yvoir, Belgium; 4 Department of Chemistry & Institute of Nanotechnology & Advanced Materials (BINA), Bar-Ilan University, Max & Anna Web Street, 5290002 Ramat-Gan, Israel

**Keywords:** Functional tungsten disulfide nanotubes, Safety, Hemocompatibility, Thrombin generation

## Abstract

Inorganic transition metal dichalcogenide nanostructures are interesting for several biomedical applications such as coating for medical devices (e.g. endodontic files, catheter stents) and reinforcement of scaffolds for tissue engineering. However, their impact on human blood is unknown. A unique nanomaterial surface-engineering chemical methodology was used to fabricate functional polyacidic polyCOOH inorganic nanotubes of tungsten disulfide towards covalent binding of any desired molecule/organic species via chemical activation/reactivity of this former polyCOOH shell. The impact of these nanotubes on hemolysis, platelet aggregation and blood coagulation has been assessed using spectrophotometric measurement, light transmission aggregometry and thrombin generation assays. The functionalized nanotubes do not induce hemolysis but decrease platelet aggregation and induce coagulation through intrinsic pathway activation. The functional nanotubes were found to be more thrombogenic than the non-functional ones, suggesting lower hemocompatibility and increased thrombotic risk with functionalized tungsten disulfide nanotubes. These functionalized nanotubes should be used with caution in blood-contacting devices.

## Introduction

Inorganic transition metal dichalcogenide (TMD) materials, such as tungsten and molybdenum disulfides (WS_2_ and MoS_2_, respectively) are of significant interest to the scientific community because of their unique multi-layered structures and functional properties, with nano-sized fullerene-like (IF) particles tending to exhibit a different set of properties compared to the corresponding bulk forms. These metal dichalcogenide nanomaterials have emerged as one of the most promising classes of nanomaterials since the discovery of carbon nanotubes (CNTs) [1–8]. As with early researches in the field of CNTs, a wide number of potential applications have been proposed and investigated including areas such as energy storage [9], field effect transistors [10], nanocomposite coatings [11, 12], battery anodes [13], light-emitting diodes [14], self-lubricating medical devices [15], and high-performance nanoscale lubricants [16–23]. In addition, the outstanding shock absorbing ability of IFs-WS_2_ nanotubes holds a great potential for new impact and shock-resistant materials [24–26]. Composite hybrid materials formed by incorporating small amounts (less than 5% weight ratios) of such nano-sized inorganic fillers into any given polymer matrix are also of particular interest, showing improved mechanical properties, higher thermal properties, and improved performances as barriers to heat, moisture, and solvents [27–29] when compared to similar composites prepared with conventional fillers [28, 30]. Indeed, considerable research work has been conducted dealing with polymer-based nanocomposites that incorporate inorganic IFs-WS_2_ NPs into matrices of epoxy [30], polystyrene/poly(methylmethacrylate) [28], poly(propylene fumarate) [29], nylon 12 [31], and poly(phenylene) sulphide [32]. Due to the superior mechanical properties of corresponding inorganic IFs-WS_2_ NPs, such as high stiffness and strength [33], ultrahigh-performance polymer nanocomposites have been readily produced [34]. In addition, commercial performant lubricants are now presently available that include same inorganic IFs-WS_2_ NPs that impart unique tribological properties [35] to the corresponding final composite products. Although there are many potential applications in a wide variety of fields for such inorganic metal dichalcogenide IFs-WS_2_ NPs and inorganic INTs-WS_2_ nanotubes (INTs), novel developmental research has been strongly hampered analogously to early CNTs-based research. Indeed, these dichalcogenide nanomaterials are highly hydrophobic, thus quite insoluble in common organic/aqueous solvents, difficult to homogeneously disperse into most liquids and resins, while disclosing serious limited dual phase compatibility when admixed with common polymers.

**Fig. 1:**
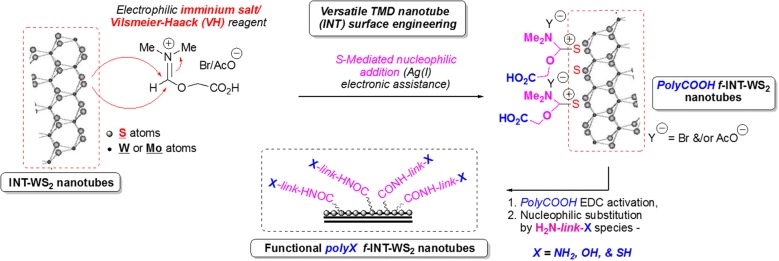
Preparation of functional polyX (X: COOH, NH_2_, OH, SH) *f*-INT-WS_2_ inorganic nanotubes. The functional inorganic nanotubes were prepared using electrophilic VH complex and subsequent covalent chemical derivatizations

In this specific context, we recently developed a unique nanomaterial surface-engineering chemical methodology to fabricate covalently decorated functional polyacidic polyCOOH–INTs-WS_2_ using Vilsmeier–Haack (VH) complex chemistry/reactivity (polyCOOH shell decoration) [36]. This novel surface engineering method enables effective covalent bonding of any desired molecule/organic species via polyCOOH shell chemical activation/reactivity that may improve and optimize any requested interfacial property of corresponding functional INTs-WS_2_ (*f*-INTs-WS_2_). This polycarboxylated shell can be readily exploited as an anchoring shell for subsequent second-step covalent attachment of a wide variety of organic molecules/polymers, including even other components such as NPs, for example, onto the functional nanotube surface. Therefore, a quite versatile simple organic activation chemistry (EDC•HCl activation of polyCOOH shell/species) readily enables corresponding surface property tuning to match those requested for any contacting material (polymeric phases, solvents, etc.). Moreover and in this context, by employing appropriate bifunctional linkers such as those described in this study (obtainment of novel 2nd step polyNH_2_/polySH/polyOH shells, Fig. 1), the resulting chemically modified *f*-INT-WS_2_ can be covalently bound to an even wider variety of reactivity-complementing materials.

Recent progress in studies of this original novel class of inorganic nanomaterials suggests that they can be also impregnated into metallic coatings for medical administration/application [37]. For example, it was demonstrated that the use of orthodontic wires coated with metallic films containing IFs-WS_2_ NPs in dentistry could significantly reduce the mechanical forces required for teeth realignment, thus preventing unnecessary excess forces that would lead to unacceptable teeth movement, longer treatment, and adverse damage to the roots of the teeth [10, 37, 38].

Since both IFs-WS_2_ NPs and INTs-WS_2_ are already commercially available in the market thus providing effective potentialities of incorporation/involvement towards innovative future medical applications, extensive research investigations concerning the overall biocompatibility and toxicity of these inorganic materials need to be performed to ensure that they are safe for composite-based usage. Researches on the toxicity of TMD nanomaterials is still in its infancy with only a handful of assessments performed on IFs-MoS_2_ and IFs-WS_2_ NPs. Preliminary results from in vivo toxicology tests of IFs-WS_2_ NPs showed no apparent toxic effects on mammals, suggesting its high biocompatibility [39]. In addition, in vitro cytotoxicity examination of IFs-MoS_2_ NPs on three different human cell lines (i.e. CCC-ESF-1, A549, and K562) revealed that they are nontoxic to cells after 48 h exposure [17]. However, at the present time, no experimental studies assessed the hemocompatibility of TMD materials. With the influx of research and possible commercialization of TMDs in the future, it is vital to both initiate hemocompatibility studies of this group of nanomaterials and assess their impact on hemolysis, platelet functions, and blood coagulation [40].

In this special work, we characterized the hemocompatibility of such different functional INTs-WS_2_ and assessed their impact on red blood cells, platelet aggregation and blood coagulation using human blood.

## Methods

### Materials

Non-functional INTs-WS_2_ have been bought from NanoMaterials Ltd. Company (Yavne, Israel). All reagents and solvents have been purchased from commercial sources and used without any further purification. Thermogravimetric analyses (TGA) have been performed on a TA Q600-0348, model SDT Q600 (Thermofinnigan) device using a temperature profile of 25–800 °C at 10 °C/min under nitrogen flow (180 mL/min) with sample amounts of 5–15 mg. Infrared (IR) spectra were recorded on a Fourier transform infrared spectrometer Tensor 27 (Bruker) using attenuated total reflectance (ATR). Nanomaterial surface charges were evaluated by ξ potential measurements using a Zetasizer Nano-ZS device (Malvern Instruments Ltd., United Kingdom) in water (pH adjusted) at 25 °C and 150 V. Both VH-untreated starting and resulting VH-modified f-INTs-WS_2_ nanotubes have been also characterized using G2, FEI High Resolution transmission electron microscopy (TEM) (Tecnai). Dispersions of INT-WS_2_ and *f*-INT-WS_2_ have been prepared with a low-power ElmaSonic S30 bath sonicator (Elma GmbH & Co., Deutschland). The chemically accessible polyCOOH shell present on the surface of the polyCOOH *f*-INT-WS_2_ has been also quantified by both (i) Kaiser testing after shell derivatization using 1,3-diaminopropane and (ii) Ellman’s one after subsequent similar shell derivatization using cysteamine.

### Polycarboxylation of INT-WS_2_—fabrication of polyCOOH-*f*-INT-WS_2_

To a solution of 2-bromoacetic acid (2-BrCH_2_COOH), (1.0 g, 7.19 mmol) in anhydrous dimethyl formamide (DMF, 3 mL) was added Ag(I)OAc (10.0 mg, 0.059 mmol) and dry INT-WS_2_ (200.0 mg). The mixture was heated in an oil bath to 80 °C and stirred over 2 days at the same temperature. After cooling to room temperature, the mixture was centrifuged (11,000 rpm, 5 min). The resulting cleaned (EtOH, 5 washing cycles) solids were dried under vacuum to obtain 190 mg of functional polyCOOH *f*-INT-WS_2_.

### Diamine coupling onto polyCOOH *f*-INT-WS_2_—fabrication of polyNH_2_-*f*-INT-WS_2_

To a solution of 1-ethyl-3-(3-dimethylaminopropyl)carbodiimide (EDC, 20.0 mg, 4 mmol) in dichloromethane (DCM, 12 mL) was added polyCOOH *f*-INT-WS_2_ (200.0 mg) and 4-dimethylaminopyridine (DMAP, 10.0 mg, 0.08 mmol). The mixture was stirred for 2 h at room temperature followed by addition of 1,3-diaminopropane (NH_2_–(CH_2_)_3_–NH_2_, 800 µL, 9.58 mmol) and stirring continued at room temperature overnight. The mixture was centrifuged (11,000 rpm, 5 min) and the supernatant discarded. The solids were worked up as described for former polyCOOH *f*-INT-WS_2_. The product contained 0.77 mmol NH_2_ groups/g of polyNH_2_ *f*-INT-WS_2_ as determined by Kaiser testing.

### Cysteamine coupling onto polyCOOH *f*-INT-WS_2_—fabrication of polySH-*f*-INT-WS_2_

To a solution of EDC (3.0 g, 19.32 mmol) in DCM (40 mL) was added polyCOOH *f*-INT-WS_2_ (1.8 g). The suspension was stirred for 2 h at room temperature followed by addition of cysteamine (NH_2_–(CH_2_)_2_–SH, 4.0 g, 51.85 mmol) and DMAP (20.0 mg, 0.16 mmol) and stirring continued for 2 days at room temperature. The mixture was centrifuged (11,000 rpm, 5 min) and the supernatant discarded. The solids were worked up as described for former polyCOOH *f*-INT-WS_2_ to obtain 1.6 g of functional product. The product contained 0.8 mmol SH groups/g of polySH *f*-INT-WS_2_, as determined by Ellman testing.

### 2-Aminoethanol coupling onto polyCOOH *f*-INT-WS_2_—Fabrication of polyOH-*f*-INT-WS_2_

To a solution of EDC (3.0 g, 19.32 mmol) in DCM (40 mL) was added polyCOOH *f*-INT-WS_2_ (1.5 g). The suspension was stirred for 2 h at room temperature followed by addition of 2-aminoethanol (NH_2_–(CH_2_)_2_–OH, 4.0 mL, 64.71 mmol) and DMAP (20.0 mg, 0.16 mmol) and stirring continued for 2 days at room temperature. The mixture was centrifuged (11,000 rpm, 5 min) and the supernatant discarded. The solids were worked up as described for former polyCOOH *f*-INT-WS_2_ to obtain 1.3 g of functional product.

### Preparation of human platelet-rich plasma, platelet-poor plasma, normal pooled plasma and washed red blood cells suspension

Human platelet rich plasma (PRP), platelet poor plasma (PPP), whole blood, washed red blood cell (RBC) suspension and normal pool plasma (NPP) were prepared with blood from healthy volunteers who were free from any medication for at least 2 weeks. Blood was collected by venipuncture into tubes containing buffered sodium citrate (109 mM, nine parts blood to one part of sodium citrate solution) (BD Vacutainer^®^). The study protocol was in accordance with the Declaration of Helsinki and was approved by the Medical Ethical Committee of the CHU UCL Namur (Yvoir, Belgium).

PRP was carefully prepared by centrifugation at 200*g* of whole blood at room temperature for 10 min. The platelet count was adjusted to 300,000 platelets/μL and PRP was used immediately after preparation. Platelet free plasma used to adjust platelet concentration is obtained after centrifugation at 2000*g* in 10 min of the pellet at room temperature.

The preparation of washed RBC suspension was prepared by centrifugation of whole blood at 3000*g* over 5 min. The PPP is removed and used for interference assays. RBC are washed with physiological phosphate buffered saline (PBS, 6.7 mM phosphate, pH = 7.4) three times with intermediate centrifugation of 3000*g* over 5 min. RBC are then resuspended in PBS with the same volume as PBS removed.

For NPP, a total of 47 healthy individuals were included in the study. The exclusion criteria were thrombotic and/or hemorrhagic events, antiplatelet and/or anticoagulant medication, pregnancy and uptake of drugs potentially affecting the platelet and/or coagulation factor functions during the 2 weeks prior to the blood drawn. A written informed consent was obtained from each donor. The study population displayed the following characteristics: 27 females and 20 males aged from 18 to 53 years (mean age = 25 years) with body mass index (BMI) ranging from 17.6 to 34.9 kg/m^2^ (mean BMI = 22.7 kg/m^2^). After collection of blood, the PPP was obtained from the supernatant fraction of the blood tubes after a double centrifugation for 15 min at 2000*g* at room temperature. It was immediately frozen at − 80 °C after centrifugation. The NPP samples were thawed and kept at 37 °C just before use.

### Hemolysis assays

Hemolysis assays were performed as previously described on the blood of one healthy donor [41]. Briefly, 15 μL of nanomaterial suspended in tyrode, tyrode (negative control) or triton X-100 (positive control) are added to 285 μL of whole blood or washed RBC (final NP concentration: 100 µg/mL). The suspension is incubated at room temperature on a shaking plate during 1 h. After the incubation time, the suspension is centrifuged at 10,000*g* over 5 min. Supernatant is read in a 96-well plate using a microplate scanning spectrophotometer XMark (Biorad, USA) at 550 nm. The percentage hemolysis was then calculated as:

$$ H \left( \% \right) = \frac{{ \left( {OD_{sample } {-} OD_{tyrode} } \right)}}{{\left( {OD_{Triton X - 100 \,at\,\, 1\% } - OD_{tyrode} } \right)}} \times 100. $$

For each term of the equation, the corresponding interference was subtracted. The interference corresponds to the same conditions except that the solution does not contain RBCs. Positive (triton X − 100 at 1%) and negative (Tyrode) controls induced 100% and 0% of hemolysis, respectively. The results were expressed as mean ± SD (n = 3).

### Light transmission aggregometry

The impact of *f*-INTs-WS_2_ on induced platelet aggregation was studied using the chronometric aggregometer type 490-2D as previously reported [41]. Briefly, the reaction mixture for induced aggregation tests contained 213 or 233 μL of PRP at 300,000 platelets/μL, with respectively 25 μL of collagen (final concentration: 190 μg/mL, calf skin, Bio/Data corporation, USA) or 5 μL of arachidonic acid (AA, final concentration: 600 μM, Calbiochem, Germany) and 12.5 μL of NPs at final concentration of 100 μg/mL. Inducers alone were also used before any experiment to check platelet reactivity. PPP was used as a reference. Data were collected with the chronolog two channel recorders at 405 nm connected to a computer.

### Coagulation: calibrated thrombin generation test (cTGT)

The impact of non-functional and functional INTs-WS_2_ on coagulation was studied using the calibrated thrombin generation test (cTGT) as previously reported [41]. For each experiment, a fresh mixture of fluorogenic substrate/calcium chloride buffered solution was prepared as follows: 2.6 mL of Fluo Buffer^®^ (Thrombinoscope BV, The Netherlands) were mixed with 65 μL of Fluo substrate^®^ (100 mM in DMSO, Thrombinoscope BV, The Netherlands). PPP-Reagent, PPP-Reagent LOW, MP-Reagent and Thrombin Calibrator (Thrombinoscope BV, The Netherlands) are four inducers, giving final assay concentrations of 5 pM tissue factor (TF) with 4 μM phospholipids (PL) and 16.7 mM CaCl_2_; 1 pM TF with 4 μM PL and 16.7 mM CaCl_2_; 4 μM PL and 16.7 mM CaCl_2_; and 620 nM α2- macroglobulin-thrombin complex, respectively. They are reconstituted with 1 mL distilled water according to the instructions provided by the manufacturer. A calibration curve was simultaneously performed using the thrombin calibrator. The acquired data were automatically processed by the software, which provided thrombin activity curves and 3 parameters based on this curve: lagtime (minutes), peak concentration (nM) and endogenous thrombin potential (ETP, nM × minutes). The INT/*f*-INTs suspensions were tested at final concentrations from 5 to 500 μg/mL. Statistical analyses were conducted with an unpaired t-test using the GraphPad Prism software (GraphPad software, v 5.01, USA).

## Results

### Fabrication and characterization of *f*-INTs-WS_2_

Functional INTs-WS_2_ have been effectively fabricated using the two-step surface engineering methodology described in Fig. 1 below. First and as the first critical chemical modification methodology, a strongly electrophilic VH complex arising from DMF–BrCH_2_COOH reactivity has been generated in situ in the presence of starting INTs-WS_2_ to provide intermediate polyacidic functional polyCOOH *f*-INTs-WS_2_.

In a 2nd derivatization step, resulting chemically modified polyCOOH *f*-INTs-WS_2_ nanotubes might be readily chemically activated (EDC activation) and reacted with bifunctional nucleophilic linkers of the type **H**_**2**_**N-link-X** to provide corresponding functional **polyX** (polyNH_2_, polySH, polyOH) *f*-INTs-WS_2_ nanotubes. All these functional nanomaterials have been fully characterized by combined thermogravimetric analysis (TGA), spectroscopic FT-IR/XPS, XRD, Kaiser (NH_2_ species quantification)/Ellman (SH species quantification) tests, HR-TEM and ζ potential values measurements (Table 1). All these characterization spectroscopy-based spectra/data and TEM/HR-TEM microphotographs including nanomaterials are fully detailed in the corresponding Ref. [36].

**Table 1:** Selected characterization (TGA) and functionality quantification data

Material	Kaiser test (mmol/g)	Ellman’ s test (mmol/g)	TGA—% weight loss (25–800 °C range)	ζ potential value (mV)
INTs-WS_2_	–	–	~ 3%	− 25.0
INTs-WS_2_COOH	–	–	11%	− 34.7
INTs-WS_2_NH_2_	0.77	–	19%	− 18.9
INTs-WS_2_SH	–	0.8	14%	− 28.4
INTs-WS_2_OH	–	–	12%	− 27.2
INTs-WS_2_OH: specific characterizing IR data	[2683–3190–3525 cm^−1^]: O–H stretchings set (OH organic species); 1620 and 1520 cm-1: C=O stretchings of carbonyl and amide species; 1520 cm^−1^: C–H stretchings (saturated aliphatic species)			

INTs, inorganic nanotubes; TGA, thermogravimetric analysis—starting INTs-WS_2_ nanotubes are negatively charged (− 25.0 mV) due to known OH-based defects arising from industrial nanofabrication step

### Hemocompatibility

#### Red blood cells

**Fig. 2:**
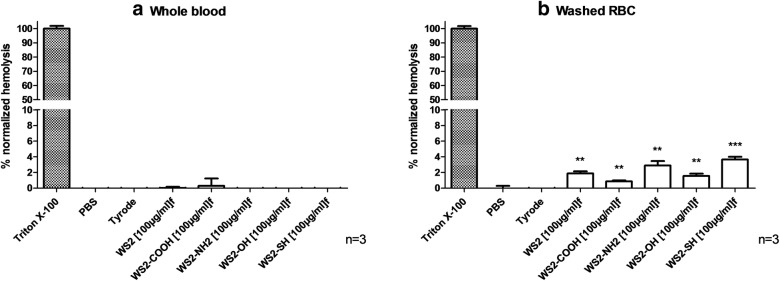
Impact of INTs-WS_2_/*f*-INTs-WS_2_ on hemolysis after 1 h at 100 μg/mL. Experiments were performed on **a** whole blood and **b** washed RBC. Triton X-100 1% and Tyrode buffer (v/v) were respectively used as positive and negative controls. Mean (%) ± SD, n = 3

Absorbance spectrum of RBC suspension 10% (v/v) supernatant incubated with Triton X-100 1% (v/v) is measured. The interference of nanotubes within assay is determined at 550 nm. This interference was avoided by subtracting the OD_550_ nm of INTs-WS_2_/*f*-INTs-WS_2_ suspended in the vehicle from the measured OD_550_ nm at the same concentration (data not shown). Measurement of absorbance at 550 nm in whole blood or washed RBC supernatant assesses the release of hemoglobin from lysis RBCs. Both non-functionalized and functionalized INTs-WS_2_/*f*-INTs-WS_2_ at 100 µg/mL did not induce hemolysis in whole blood (Fig. 2a) and in washed red blood cells (Fig. 2b) according to the ASTM E2524-08 standard (hemolysis ratio of all samples was below 5%) [42].

#### Platelet function

**Fig. 3:**
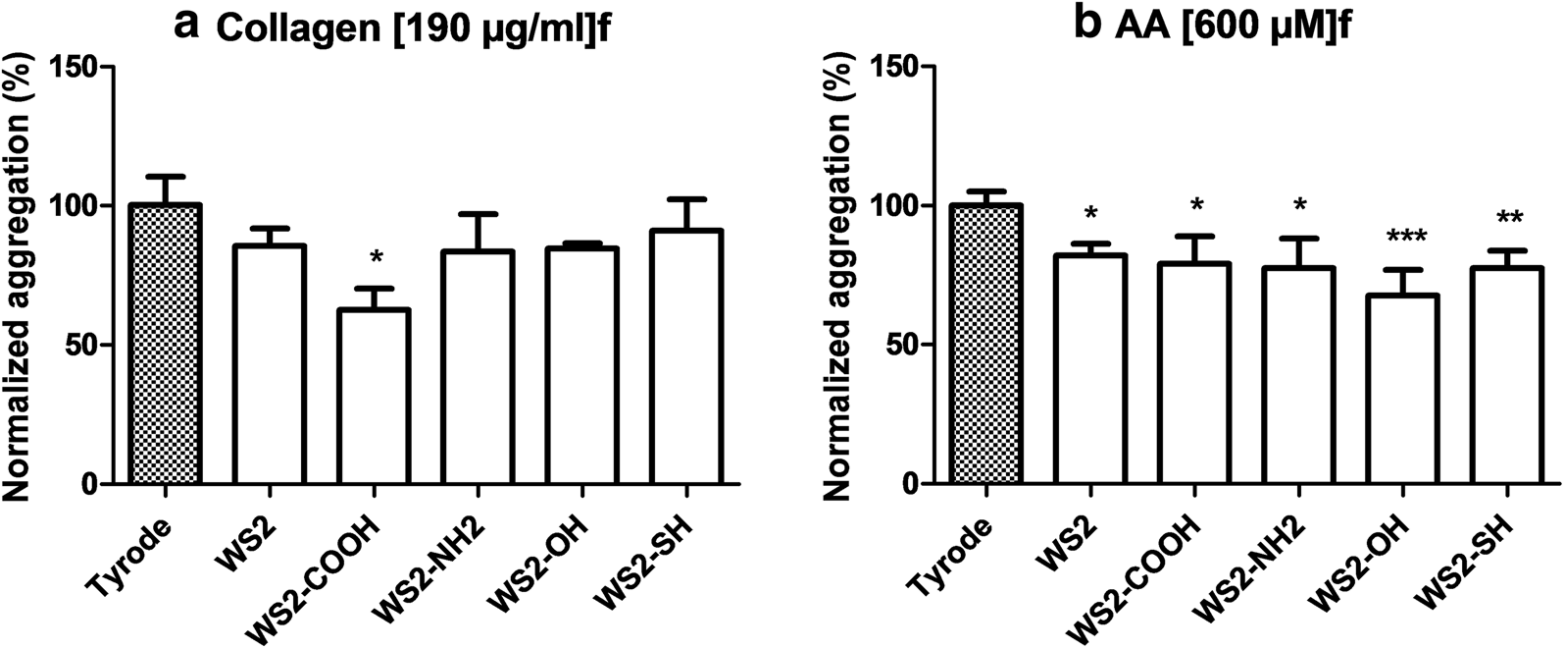
Effect of functionalized INTs-WS_2_ at 100 µg/mL on platelet aggregation. Platelet aggregation was induced by (**a**) collagen or (**b**) AA. Tyrode was used as a negative control. Results are expressed as % of response (Mean ± SD, n = 2–4)

Second important parameter to be determined is the impact on platelet and in particular on platelet aggregation. At 100 µg/mL, non-functionalized and functionalized INTs-WS_2_/*f*-INTs-WS_2_ significantly decreased platelet aggregation induced by AA (Fig. 3b). When collagen is the inductor, only polyCOOH-*f*-INTs-WS_2_ decreased significantly platelet aggregation (Fig. 3a).

#### Coagulation

**Fig. 4:**
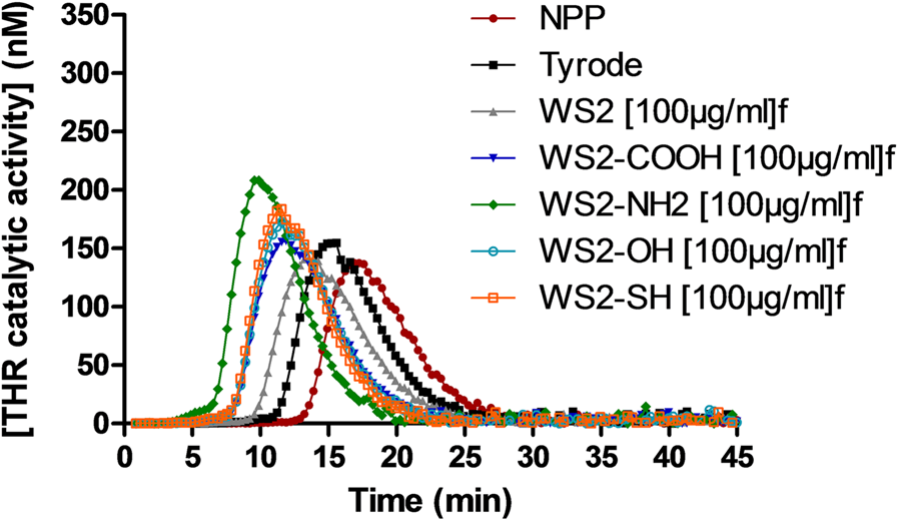
Thrombin activity profiles in the presence of INTs-WS_2_/*f*-INTs-WS_2_ at 100 µg/mL. Data represent the mean of three independent experiments

Impact of *f*-INTs-WS_2_ on blood coagulation was assessed through cTGT. Non-functionalized and functionalized INTs-WS_2_/*f*-INTs-WS_2_ impact blood coagulation when the intrinsic pathway is triggered **(**Fig. 4). A procoagulant effect of these nanomaterials is observed with a decrease of lagtime and an increase of peak concentration and ETP (Table 2). Based on their procoagulant activity on the intrinsic pathway, INTs-WS_2_/*f*-INTs-WS_2_ can be classified as follows: WS_2_-NH_2_ > WS_2_-OH > WS_2_-SH=WS_2_-COOH > WS_2_. Experiments with coagulation initiated by the extrinsic and common pathways demonstrated no effect of *f*-INTs-WS_2_ at the exception of polyNH-*f*-INTs-WS_2_ which had a procoagulant effect when common pathway is triggered (data not shown).

**Table 2:** Influence of INTs-WS_2_/*f*-INTs-WS_2_ at 100 µg/mL on thrombin generation parameters induced by the intrinsic pathway

4 µMPL	% Lagtime	% Lagtime SD	% ETP	% ETP SD	% Peak	% Peak SD
NPP	100	9	100	9	100	11
Tyrode	81	1	110	4	112	6
WS2	71	5	107	3	102	3
WS2-COOH	58	2	114	3	113	2
WS2-NH2	48	3	130	5	150	19
WS2-OH	59	3	119	6	122	10
WS2-SH	58	2	119	1	131	6

ETP, endogenous thrombin potential; NPP, normal pool plasma

Data are expressed in percentage in comparison with control (PBS) (n = 3)

## Discussion

As quite novel inorganic multi-layered nanomaterials, hydrophobic non-functional INTs-WS_2_ nanotubes have been recently shown to be reactive towards a strongly electrophilic acidic VH complex arising from both DMF/Br-CH_2_COOH reagents that enabled stable covalent nanotube surface chemical engineering/chemical modification by a corresponding polyCOOH shell (polyCOOH *f*-INTs-WS_2_ nanotubes). Quite innovatively while using specific bifunctional linkers (Fig. 1), this polyacidic shell might be readily exploited via EDC activation for additional surface engineering to get a wide variety of functional *f*-INTs-WS_2_ inorganic nanotubes, i.e., polyNH_2_/polySH/polyOH *f*-INTs-WS_2_ nanotubes [36]. It must be noticed that this innovative covalent surface engineering enables the quite effective development of any requested appropriate interfacial surface feature (surface reactive functionality, surface hydrophobicity/hydrophilicity balance) when incorporated into any polymeric matrix for example.

Before being used in human, biocompatibility of blood contacting devices needs to be considered to detect potential deleterious effects. Cytotoxicity studies have been initiated with TMD nanomaterials and first results are encouraging. In vitro studies have been conducted in different cellular models and do not demonstrate WS_2_ nanotubes induced cytotoxicity [43, 44]. Teo Chng confirmed this safety profile and demonstrates that WS_2_ is the least toxic of TMD nanomaterials [45]. In vivo studies in murine models confirmed the safety of these particles [46, 47]. In addition to cytotoxicity studies, hemocompatibility assays are also part of preclinical assessment of any biomedical device according to ISO-10993-4. Common hemocompatibility testing includes hemolysis, platelet function, and coagulation assays. The hemocompatibility of TMD is to our knowledge currently unknown. For the first time, we are reporting here the impact of non-functional/functional INTs-WS_2_/*f*-INTs-WS_2_ on human blood. Additionally, physicochemical properties of nanomaterials (e.g. NP shape, hydrophilicity, solubility, size, chemical composition) are linked to toxic outcomes. As a matter of direct consequence, it has been quite attractive to determine, check, and eventually confirm how such versatile surface engineering functionalization shells might influence the hemocompatibility of corresponding surface-engineered INTs-WS_2_.

Hemolysis refers to the destruction of red blood cells inducing release and buildup of toxic red blood cell content (i.e. hemoglobin), which may cause potential life-threatening conditions (e.g. hepatic and renal injuries). Because of their small size, nanomaterials bind red blood cells and could induce by this way hemolysis [48]. Therefore, assessment of hemolytic potential of all medical devices in contact with blood is required. We assessed the hemolytic potential of our nanomaterials using a spectrophotometric assay suitable to study of nanomaterials (i.e. nanoparticle/nanotube interferences need to be ruled out) [49] and demonstrated that non-functionalized and functionalized INTs-WS_2_/*f*-INTs-WS_2_ do not impact hemolysis on human blood and washed red blood cells (i.e. results below the 5% threshold) in accordance to ISO-10993-4. Higher levels of hemolysis are reported in experiments with washed red blood cells compared to those performed in whole blood. This difference was previously reported with silver and silica nanoparticles and is possibly related to the adsorption of human plasma biomolecules on nanoparticles, which possibly affect their hemolytic potential [41, 50]. Our results are in accordance with prior studies, which demonstrated no hemolytic effect of other TMD nanomaterials (i.e. MoSe_2_ nanosheets) [51, 52]. Li et al. [53] demonstrated that coating of TiNi alloy with tungsten nanomaterial reduces hemolysis rate, which confirms the safety of such materials toward red blood cells [54]. Our results are also in accordance to prior studies that indicate that nanomaterials with anionic surface does not induce hemolysis [40]. The few effect of these nanotubes on red blood cells is reassuring for future biomedical applications.

Platelet function is also part of preclinical characterization and is an important parameter to predict impact of nanomaterials on human blood clotting. Indeed, hemostasis is regulated by both plasmatic coagulation and platelet functions and alteration of platelet functions may lead to either bleeding or thrombosis [55]. Our study assessed platelet aggregation on human blood by light transmission aggregometry following activation by two different inducers, a suitable method to assess nanomaterial potential [56]. We demonstrate nonsignificant decrease of collagen-induced platelet aggregation by *f*-INTs-WS_2_ and also that same *f*-INTs-WS_2_ decrease platelet aggregation when induced by arachidonic acid. To our best knowledge, no other investigated impact of such nanomaterials on platelet functions has been ever reported. Therefore, the mechanism by which *f*-INTs-WS_2_ induced decreased platelet aggregation is unknown. Potential hypothesis to explain this effect on platelets could be that these nanomaterials decrease agonist-induced activation. Additionally, the hydrophobicity of functional groups might be implicated in the decreased platelet aggregation. Indeed, Elbert and Hubbell have demonstrated that hydrophobic surfaces adsorb more proteins which might cause platelet adhesion and activation and therefore be responsible of blood clot [57]. This might explain why functionalization through addition of highly hydrophilic COOH groups reduces collagen-induced platelet aggregation.

As foreign materials, biomedical devices can activate human blood coagulation and dysregulate hemostasis. Human blood coagulation is characterized by a cascade of sequential proteolytic reactions which can be initiated by two pathways, the intrinsic and extrinsic ones, that both converge to thrombin generation [55]. Because coagulation is dependent to thrombin, we studied the impact of our various nanotubes on human coagulation through a thrombin generation assay, a suitable method to assess nanomaterial impact on coagulation [58] compared to routine tests, which are insensitive for small changes [55]. An additional advantage of this test is that it is performed on human plasma, a protein-containing media which limits nanomaterial interference by their coating with physiological proteins [55]. We demonstrate that non-functional INTs-WS_2_ possess a procoagulant activity, which is accentuated by the functionalization feature of relating corresponding functional *f*-INTs-WS_2_ nanomaterials. This procoagulant effect is mediated by activation of the intrinsic pathway while INTs-WS_2_ do not affect the extrinsic pathway (data not shown). This is in line with data prior studies which indicate that nanomaterials mainly activate coagulation through intrinsic pathway [55].

The mechanism by which *f*-INTs-WS_2_ induce coagulation is unknown. Numerous nanomaterial physicochemical properties are implicated in hemocompatibility and nanomaterial surface is predominant because of its interactions with plasma proteins [59]. Zeta potential is an indicator of surface charge and has been already used to predict nanomaterial effects on human health [60]. Indeed, negatively charged surfaces are expected to be more thrombogenic because contact with anionic surface initiates physiological coagulation [61]. An hypothesis suggests that the procoagulant effect of some nanomaterials is the consequence of their binding capacity with coagulation factors which induce their activation [59]. Factor XII, a factor implicated in the intrinsic pathway, is of special interest and might undergo self-activation after interaction with an anionic surface [61]. Additionally, it was already demonstrated that anionic carbon nanotubes effectively induce human coagulation through activation of the intrinsic pathway [55]. Therefore, the anionic properties of our INTs-WS_2_ may explain their prothrombotic activity. Additionally, functionalization of our INTs-WS_2_ modifies surface properties and decreases zeta potential values, at the exception of NH_2_-INTs-WS_2_ [36]. Our study reports correlation between thrombotic potential of *f*-INTs-WS_2_ and their zeta potential, at the exception of NH_2_-INTs-WS_2_. However, surface charges are difficult to interpret because of binding of proteins on nanomaterial surface and because zeta potential was determined in protein-free media (i.e. in water) compared to coagulation testing performed in human plasma. Finally, it is interesting to highlight that in our study, TGA weight loss correlates with TGTc peak concentration, with higher weight loss and procoagulant activity with NH_2_-INTs-WS_2_. TGA determines the amount of organic material bound to the *f*-INTs [36]. Therefore and together with their unique zwitterionic surface charge features (mixed positive ammonium/NH_3_^+^ charges with negative OH-based defects), one might speculate that NH_2_-INTs-WS_2_ might better promote and bind highest amounts of organic materials to more effectively induce coagulation by better binding coagulation factors.

Tungsten disulfide nanostructures possess interesting physicochemical properties and high load bearing properties implying new opportunities in medicine [47, 62]. Potential health applications include blood-contacting and invasive devices (e.g. medical device coating, drug delivery inorganic systems, reinforcement of scaffolds for tissue engineering) [32, 46]. Moreover and quite recently, same NH_2_-INTs-WS_2_ nanomaterials have been successfully derivatized by nanotube surface-localised C-quantum dots towards both (i) cancer cell fluorescence imaging/investigation, and (ii) quite effective photothermal cell killing capability (PTT therapy potentiality), [63] thus opening a quite attractive future field of PTT cancer therapy by such non-toxic inorganic nanotubes (nanoparticle theranostics) [64, 65]. Serious concerns exist about nanomaterial-induced coagulation disorders. Therefore, the analysis of nanomaterial toxic effects on human blood cells is quite mandatory. We demonstrated using in vitro models that INTs-WS_2_ decrease platelet aggregation and induce a procoagulant state that is heighten by both functionalization type and level of innovative functional nanotubes. This observed effect on coagulation can be either beneficial or adverse according to its applications Therefore, we recommend the use of the functionalized nanoparticles in applications that imply blood coagulation such as wound dressing.

## Abbreviations

AA: arachidonic acid
ATR: attenuated total reflectance
BMI: body mass index
CNT: carbon nanotube
cTGT: calibrated thrombin generation test
DCM: dichloromethane
DMAP: 4-dimetylaminopyridine
DMF: dimethyl formamide
EDC: 1-ethyl-3-(3-dimethylaminopropyl)carbodiimide
ETP: endogenous thrombin potential
f-INT: functional inorganic nanotube
IF: fullerene-like
IR: infrared
MbS_2_: molybdenum disulfide
NPP: normal pool plasma
PL: phospholipid
PPP: platelet-poor plasma
PRP: platelet-rich plasma
RBC: red blood cell
TEM: transmission electron microscopy
TF: tissue factor
TGA: thermogravimetric analyses
TMD: transition metal dichalcogenide
VH: Vilsmeier–Haack
WS_2_: tungsten disulfide
